# Comparative analysis of two analytical methodologies for deriving dietary patterns associated with vitamin D insufficiency and anemia among expectant mothers

**DOI:** 10.7150/ijms.124006

**Published:** 2026-01-01

**Authors:** Arpita Das, Chien-Yeh Hsu, Chyi-Huey Bai, Jung-Su Chang, Ya-Li Huang, Fan-Fen Wang, Nhi Thi Hong Nguyen, Yi-Chun Chen, Jane C.-J. Chao

**Affiliations:** 1 School of Nutrition and Health Sciences, Taipei Medical University, Taipei, Taiwan.; 2 Department of Information Management, National Taipei University of Nursing and Health Sciences, Taipei, Taiwan.; 3 Master Program in Global Health and Health Security, Taipei Medical University, Taipei, Taiwan.; 4 Department of Public Health, School of Medicine, Taipei Medical University, Taipei, Taiwan.; 5 School of Public Health, Taipei Medical University, Taipei, Taiwan.; 6 Nutrition Research Center, Taipei Medical University Hospital, Taipei, Taiwan.; 7 Graduate Institute of Metabolism and Obesity Sciences, Taipei Medical University, Taipei, Taiwan.; 8 TMU Research Center for Digestive Medicine, Taipei Medical University, Taipei, Taiwan.; 9 Department of Internal Medicine, Yangming Branch, Taipei City Hospital, Taipei, Taiwan.; 10 Health Personnel Training Institute, University of Medicine and Pharmacy, Hue University, Hue, Vietnam.

**Keywords:** principal component analysis, reduced rank regression analysis, serum vitamin D, anemia-related biomarkers

## Abstract

**Objectives:** Dietary patterns play a role associated with acute or chronic diseases. This study compared the correlation between dietary patterns and vitamin D status, using two methods for dietary pattern identification, related to gestational anemia among expectant mothers.

**Methods:** In this cross-sectional study a total of 1502 expectant mothers aged > 15 years were recruited from the Nutrition and Health Survey data. Dietary patterns were discerned through principal component analysis (PCA) and reduced rank regression (RRR). Associations between dietary pattern scores, vitamin D, and anemia-related biomarkers were validated using linear and binomial logistic regression adjusted for sociodemographic and economic factors.

**Results:** Two dietary patterns were identified: a plant and marine-based dietary pattern (PMDP) via PCA and a convenience food dietary pattern (CFDP) via RRR. PMDP was characterized by high intakes of plant and marine foods, while CFDP showed high intakes of canned and pickled foods. Risk estimation indicated that high PMDP intake was associated with 30% reduction in odds of vitamin D insufficiency, while moderate CFDP intake was linked to an increased risk of vitamin D insufficiency by 46%. Logistic regression analysis showed a positive association between CFDP and serum iron (β = 0.08; 95% CI: 0.05, 0.24), but a negative association with total iron binding capacity (β = -0.05; 95% CI: -0.44, -0.07).

**Conclusions:** The PCA-derived dietary pattern effectively identifies eating patterns, while the RRR-derived dietary pattern has a better estimate for disease associations in a specific population. The RRR-derived dietary pattern provides valuable insights for dietary guidelines targeting vitamin D insufficiency.

## Introduction

Vitamin D deficiency and insufficiency have become a global health concern, and referred as “vitamin D deficiency epidemic” 1. The prevalence of these conditions was widespread, with 15.7%, 47.9%, and 76.6% of individuals having serum 25(OH) vitamin D levels below 30, 50, and 75 nmol/L, respectively 2. In northern Taiwan, the previous study found that 30.1% of pregnant women had low serum vitamin D levels (< 50 nmol/L) 3. Vitamin D_3_, or calcitriol, plays a vital role in preventing metabolic diseases like rickets, osteomalacia, type 2 diabetes, cardiovascular disease, cancer, and chronic kidney disease 4. During pregnancy, vitamin D supplementation has been linked to better maternal and fetal outcomes, including preeclampsia, gestational diabetes, improved fetal growth, preterm birth, and reduced risk of small for gestational age (SGA) 5.

Serum hemoglobin levels were positively correlated with serum vitamin D level in pregnant women 6, and the meta-analyses reported a 61% increased risk of anemia in pregnant women with vitamin D deficiency (OR = 1.61; 95% CI: 1.41, 1.83) 7. Gestational anemia could lead to preterm delivery, SGA, reduced birth weight, low Apgar (appearance, pulse, grimace, activity, respiration) scores, and fetal complications 8. Research by Bano et al. suggested that 50% of gestational anemia cases were due to insufficient iron intake or storage 9. Factors like inadequate dietary diversity, consumption of tea or coffee with meals, insufficient meals, and multiparity increased anemia risk, while pregnant women consuming dairy, animal protein, and vitamin A-rich fruits and vegetables were associated with a reduced risk of anemia 10-13. Our previous study in Taiwan also found positive associations between dietary patterns (DPs), such as plant-based, carnivore, dairy, and non-dairy DPs, and serum vitamin D, folate, and vitamin B_12_ levels 14. Additionally, an adequate DP and frequent vitamin D supplementation were positively associated with serum ferritin and vitamin D levels 15.

Understanding DPs is essential for analyzing the complex relationships between diet and disease outcomes, as it offers a comprehensive assessment of overall dietary habits and their impact on health 16. Statistical methods like principal component analysis (PCA) and reduced rank regression (RRR) are used to identify dietary patterns in populations 17, and PCA transforms correlated food groups into uncorrelated indexes, and preserves the maximum variance of the original foods 18. However, PCA has subjectivity in selecting food groups, determining component numbers, and identifying factor loadings 18. In contrast, RRR combines both a priori and posteriori approaches, and captures foods related to disease risk and biomarkers 19-21. A previous study suggested RRR-derived DP with high intakes of plant-based and dairy products reduced the risk of micronutrient deficiencies affecting erythropoiesis in expectant mothers 22. Few studies have examined the associations of PCA- 14 or RRR-derived DP 22 with gestational anemia and vitamin D insufficiency/deficiency in expectant mothers.

Given the limited research on comparing the association of PCA- or RRR-derived DP with anemia-related biomarkers in expectant mothers, our study aimed to compare PCA- and RRR-derived DPs and evaluate their associations with anemia-related biomarkers and vitamin D levels among expectant mothers in Taiwan. Based on our previous findings, we hypothesized that RRR-derived DP might show stronger associations with anemia biomarkers and vitamin D status in expectant mothers.

## Methods

### Study population

The present cross-sectional multi-center study utilized the data from the Nationwide Nutrition and Health Survey data of Taiwanese pregnant women (NHSDT 2017-2019). A total of 1502 expectant mothers were recruited from various regions of Taiwan including the north, south, central, east, and west regions. We recruited the expectant mothers who aged > 15 years, had a maternal handbook, and signed the inform consent. The expectant mothers with multiparity > 3 or without responsiveness were excluded in this study (Supplementary Figure 1). After considering the inclusion and exclusion criteria, all participants (*n* = 1502) were categorized into two groups: (1) serum vitamin D insufficiency (≤ 75 nmol/L) (*n* = 1067) and (2) normal serum vitamin D (> 75 nmol/L) (*n* = 435). The study was conducted in accordance with the Declaration of Helsinki, and was approved by the Joint Institutional Review Board of Taipei Medical University (TMU-JIRB No. N201707039).

### Data collection

Initially, a self-reported survey was employed to collect comprehensive personal details such as socio-economic status and pregnancy-related issues. The self-reported survey included maternal demographic information such as age and region of residence and other data such as body measurements (weight and height), parity, stage of pregnancy, and sun exposure. An in-person interview by the well-trained dietitian was then carried out to determine eating habits and dietary intake. All information, including responses from the self-reported survey and dietary intake, and blood samples were collected during a single prenatal visit among expectant mothers in the first, second, or third trimester.

### Dietary assessment

Dietary data collection was conducted by referring to a validated semi-quantitative food frequency questionnaire (sFFQ) 14, 22. Food frequency was recorded as daily, weekly, or monthly consumption frequency, and consumption frequency was converted into monthly consumption frequency for the analysis of dietary patterns. A total of 59 food groups were initially identified, and 32 food groups were finally developed for the assessment of dietary patterns and the comparisons based on the nutrient compositions of the food groups 14. To mitigate recording biases, the data collection of sFFQ was assisted by registered dietitians.

### Assessment of anthropometric data

Anthropometric data, including pre-pregnancy body mass index (pBMI), were obtained through self-reported data collected during the initial trimester of pregnancy. The pBMI value was calculated by dividing the reported body weight (in kilograms) by the square of the reported height (in square meters) 23.

### Evaluation of biochemical data

For the measurement of anemia-related parameters in blood samples, approximately 8 mL of peripheral blood samples were collected during the perinatal visit. Serum, plasma, and buffy coat were segregated by centrifugation, and stored at -80°C for subsequent analysis. The process for measuring blood parameters was reported in details previously 14.

### Categorization of BMI and anemia-related biomarkers

The categorization of BMI and anemia-related biomarkers was followed the criteria demonstrated previously 14.

### Statistical methods

#### Data expression and analysis

Statistical analyses were performed using SPSS (version 22.0, IBM Corp., Armonk, NY, USA) or SAS (version 9.4, SAS Institute Inc., Chicago, IL, USA). The characteristics of the study population with insufficient or normal serum vitamin D levels were compared using Student's *t* test or chi-square test, respectively, for continuous or categorical variables. Results were presented as mean ± standard deviation. Data for binomial regression or generalized logistic regression analysis were indicated as odds ratios (ORs) and 95% confidence intervals (CIs) or β and 95% CIs, respectively, to determine the association of DP scores with vitamin D status among expectant mothers. A *p-*value < 0.05 was considered significant for all statistical analyses.

#### DP assessment

The DPs were derived using PCA and RRR methods by employing PROC FACTOR and PROC PLUS functions of the SAS program. The PCA method was employed with 32 food groups and orthogonal varimax rotation, and retained only one factor for the final comparison. The DP was developed from the RRR method using 32 food groups as predictor variables and 6 blood parameters including hemoglobin, iron, ferritin, total iron-binding capacity (TIBC), folate, and vitamin B_12_ as response variables. The first factor presenting the maximum percentage (1.70%) of variations in response variables was retained among the 6 response factors. An absolute factor loading value was set at ≥ 0.20 17 for both DPs. The DP scores for both patterns were calculated by summing up the consumption frequency score of the food groups weighed by their respective factor loading values. Based on the compositions of the DPs, PCA- or RRR-derived DP was renamed, respectively, as plant and marine-based DP (PMDP) or convenience food DP (CFDP). For further analysis, the DP scores of each DP were divided into tertiles (T) with 3 covariate-adjusted models: model 1 - unadjusted, model 2 - adjusted for age and parity, and model 3 - adjusted for age, parity, trimester of pregnancy, region of residence, and monthly family income.

## Results

### Participant characteristics

Supplementary Table 1 summarizes the general characteristics of the expectant mothers across different serum vitamin D levels. Significant differences were found in sociodemographic or pregnancy related factors, including region of residence, parity, trimester of pregnancy, and monthly family income, between the expectant mothers with normal or insufficient serum vitamin D levels. Expectant mothers with normal serum vitamin D levels were mostly resided in southern Taiwan (150 individuals, 34.6%), predominantly primiparous (213 individuals, 49.2%), in the third trimester (236 individuals, 54.3%), and had a monthly family income between NTD 30,000 and 59,999 (204 individuals, 48.0%). However, pBMI status was similar between the expectant mothers with normal or insufficient serum vitamin D levels.

### Serum biochemical characteristics of the expectant mothers across serum vitamin D levels

Table 1 summarizes serum biochemical characteristics of the expectant mothers with different levels of serum vitamin D. Participants with normal serum vitamin D levels had significantly higher serum hemoglobin (7.4 ± 1.2 g/dL), iron (14.1 ± 8.1 µmol/L), TIBC (85.4 ± 16.5 µmol/L), folate (32.5 ± 17.1 nmol/L, range: 13.5-45.3 nmol/L; 63.4%), and vitamin B_12_ (254.1 ± 178.3 pmol/L) compared to those with insufficient serum vitamin D levels.

### Dietary patterns (DPs)

Supplementary Figure 2 illustrates the dietary patterns derived from PCA or RRR analysis. The PCA-derived dietary pattern, characterized by high consumption of bamboo shoots and melons, mushrooms, roots and tubers, dark-colored vegetables, legumes, marine plants, nuts, soy products, aquatic fish, herbs and spices, fresh fruits, pickled vegetables, and salt, was named as the plant and marine-based dietary pattern (PMDP). In contrast, the RRR-derived dietary pattern was positively associated with canned and pickled fruits and jam, but negatively associated with legumes, semi-fat meat, poultry meat, lean meat, and dark-colored vegetables. This pattern was denoted as the convenience food dietary pattern (CFDP).

Notably, the dietary patterns from the two methods showed distinct characteristics. The RRR-derived dietary pattern explained a higher percentage of variation (6.55%) compared to the PCA-derived dietary pattern (4.37%). The cumulative percentage explained by RRR was approximately 1.70%, with the highest variations attributed to serum TIBC (5.11%) and iron (1.95%).

### Associations between DP scores and serum vitamin D status

Table 2 displayed the associations between the scores of DPs derived from PCA or RRR and vitamin D status. Expectant mothers exhibiting the highest intake of the PCA-derived PMDP demonstrated a reduced risk (30% to 35%) of serum vitamin D insufficiency across all three models. Conversely, expectant mothers with moderate consumption (T2) of the RRR-derived CFDP were associated with an increased risk of vitamin D insufficiency by 46% to 52% in all three models. Similar results were observed among expectant mothers with the highest consumption (T3) of the RRR-derived CFDP in models 1 and 2, with an increased risk of vitamin D insufficiency by 36% and 35%, respectively.

### Associations of PCA- or RRR-derived DP with serum anemia-related biomarkers

Table 3 presents the associations between PCA- and RRR-derived DPs and anemia-related biomarkers. Expectant mothers consuming the PCA-derived PMDP showed negative associations with serum ferritin (β = -0.06, -0.06; 95% CI: -0.28, -0.02 and -0.28, -0.02) and positive associations with TIBC (β = 0.08, 0.09; 95% CI: 0.01, 0.06 and 0.01, 0.06) in models 1 and 2.

In contrast, expectant mothers consuming the RRR-derived CFDP showed positive associations with serum hemoglobin (β = 0.06, 0.06; 95% CI: 0.00, 0.01 and 0.00, 0.01) and ferritin (β = 0.10, 0.09; 95% CI: 0.22, 0.72 and 0.20, 0.70) in models 1 and 2. Additionally, serum iron was positively associated in all three models (β = 0.10, 0.09, 0.04; 95% CI: 0.02, 0.05; 0.01, 0.05; and -0.05, 0.44) among expectant mothers with the RRR-derived CFDP. However, expectant mothers with the RRR-derived CFDP showed a negative association with serum TIBC across all three models (β = -0.17, -0.17, -0.05; 95% CI: -0.18, -0.10; -0.18, -0.10; and -0.44, -0.07).

### PCA-derived DP scores and serum anemia-related biomarkers

Table 4 shows the PCA-derived DP scores and anemia-related biomarkers. Expectant mothers in the highest tertile (T3) of the PCA-derived PMDP had 1.41 (95% CI: 1.10, 1.82) and 1.47 (95% CI: 1.13, 1.91) times higher odds of serum ferritin deficiency in models 1 and 2, respectively. Conversely, expectant mothers in the highest tertile of the PCA-derived PMDP reduced odds of serum TIBC deficiency by 34% (OR = 0.66; 95% CI: 0.50, 0.87) and 36% (OR = 0.64; 95% CI: 0.49, 0.85) in models 1 and 2, respectively.

### RRR-derived DP scores and serum anemia-related biomarkers

Table 5 displays the results for the RRR-derived dietary pattern score and serum anemia-related biomarkers. Expectant mothers with T2 of the RRR-derived CFDP score had lower odds of 0.72 (95% CI: 0.54, 0.96) and 0.74 (95% CI: 0.55, 0.99) for serum iron deficiency in both models 1 and 2, respectively. Similarly, those with T2 and T3 of the RRR-derived CFDP score had lower odds of serum ferritin deficiency (T2-model 1: OR = 0.72; 95% CI: 0.54, 0.96; model 2: OR = 0.74; 95% CI: 0.55, 1.00; T3-model 1: OR = 0.61; 95% CI: 0.45, 0.81; model 2: OR = 0.61; 95% CI: 0.46, 0.83) in both models. In contrast, expectant mothers with T2 of the RRR-derived CFDP score elevated the risk for serum TIBC deficiency by 51% (95% CI: 1.09, 2.10) and 45% (95% CI: 1.04, 2.02), and those with T3 of the RRR-derived CFDP score increased the risk for serum TIBC deficiency by 74% (95% CI: 1.26, 2.41) and 71% (95% CI: 1.23, 2.37) in models 1 and 2, respectively.

## Discussion

### Comparisons between PCA- and RRR-derived DPs

Results from the present study revealed that the PCA-derived PMDP was associated with a decreased risk of low serum vitamin D status. Conversely, the RRR-derived CFDP showed an increased risk for low serum vitamin D status. Comparing the PCA- and RRR-derived DPs, PCA analysis elucidated dietary behaviors within the population, while RRR primarily focused on outcome variables, revealing the associations with disease-related variables or biomarkers 24. Consequently, both approaches identified different DPs. The PCA-derived DP was characterized by higher intakes of plant foods, fish, pickled vegetables, and salt. In contrast, the RRR-derived DP was characterized by an increased intake of processed foods and decreased consumption of dark-colored vegetables, lean or semi-fat meat, and legumes and various beans. Consequently, the PCA-derived DP exhibited a negative association with ferritin, whereas the RRR-derived DP showed positive associations with serum anemia-related biomarkers such as hemoglobin, iron, and ferritin. These results highlighted the distinct characteristics of RRR (disease-associated) 25 and PCA (eating pattern-associated) 26 methods. Previous studies have generally reported similar DPs derived from both PCA and RRR methods 17, 27, though some studies have shown contrasting results like ours 24, 28, 29.

### PCA- or RRR-derived DP, anemia-related biomarkers, and vitamin D status

Regarding the association of DPs with serum anemia-related biomarkers and vitamin D status, our study found that the PCA-derived DP contained the hindering food groups for iron absorption, potentially explaining an inverse association with ferritin 14. Additionally, the presence of vitamin D-enriched food items in the PCA-derived DP (such as mushrooms, marine plants, and soy products) could contribute to improved vitamin D status, consistent with the previous research 14, 30-33. Furthermore, our findings were consistent with other studies indicating that traditional DP with increased consumption of rice, pork, and vegetables 34, consumption of non-heme iron and iron-inhibiting foods (such as coffee, soft drinks, and sugar-sweetened beverages) 35, fast food DP 36, and inflammatory DP (comprising of eggs and animal products in combination with processed, fried, and sweetened foods) 37 could increase the risk of anemia across various populations.

Surprisingly, we observed a positive association between RRR-derived CFDP with high intakes of canned and pickled fruits and jam and serum ferritin levels, possibly attributed to the improvement in gut microbiota profile. Pickles, in particular, are rich sources of beneficial gut bacteria, which could enhance gut microbial functions 38. This finding aligns with the report indicating that intestinal gut microbiota played a crucial role in iron absorption 39. Furthermore, we noted an inverse association between ferritin and TIBC status, consistent with previous research highlighting TIBC as a biomarker inversely related to iron status 40, 41. While the association between serum vitamin D and ferritin status remains controversial 7, 42-45. An inverse association observed between serum vitamin D and ferritin levels could be explained by the opposite properties of vitamin D as an anti-inflammatory role and ferritin as an inflammatory biomarker 46. However, vitamin D could reduce inflammatory cytokines and hepcidin levels to ensure sufficient iron storage 47. In the present study, an inverse association between serum vitamin D and ferritin status was not found in the completed adjusted model. A previous study reported that serum vitamin D was positively associated with serum hemoglobin levels, but the role of DP was not correlated with this association among women of reproductive age 48.

### Strengths and limitations

To our knowledge, the present study was the first research to explore two different statistical methods (PCA and RRR) deriving DPs, and examine the associations of DPs with serum vitamin D and anemia-related biomarkers among expectant mothers. A major strength of this study was to use the nationwide nutrition survey data, cluster sampling, and covariate-adjusted models to identify the most relevant associations, along with validated sFFQ 28. The large sample size accurately represented the characteristics of the population. However, despite these strengths, this study has several limitations, including the absence of data analysis for 24-hour dietary recall and dietary supplements, and missing information on inflammatory biomarkers, parathyroid hormone, hepcidin, and pregnancy-related eating disorders (such as pica and morning sickness).

## Conclusions

In summary, PCA- and RRR-derived DPs were compatible. The PCA-derived DP depicted population eating behavior, while the RRR-derived DP identified diet-disease associations. The PCA-derived plant and marine-based DP with high intakes of plant foods, nuts, fish, and seafood products was positively associated with serum vitamin D, but not correlated with serum anemia-related biomarkers. The RRR-derived convenience food DP with high consumption of canned or pickled fruits and jam showed negative associations with serum vitamin D and TIBC, but a positive association with serum iron. The RRR-derived DP may provide the insight for dietary guidelines targeting vitamin D insufficiency. Further research should explore DP scores and gestational anemia risk, incorporating additional anemia and vitamin D biomarkers.

## Supplementary Material

Supplementary figures and table.

## Figures and Tables

**Table 1:** Serum biochemical characteristics of expectant mothers across different levels of vitamin D

Serum variables	Total population (*n* = 1502)	Insufficient vitamin D ≤ 75 nmol/L (*n* = 1067)	Normal vitamin D > 75 nmol/L (*n* = 435)	*p*
Hemoglobin, mmol/L, *n* (%)	7.3 ± 1.2	7.2 ± 1.2	7.4 ± 1.2	0.017
< 4.34	10 (0.7)	9 (0.9)	1 (0.2)	0.217
4.34-6.21	123 (8.2)	87 (8.2)	36 (8.3)	
6.21-6.83	189 (12.6)	141 (13.2)	48 (11.0)	
> 6.83-8.69	1108 (73.8)	785 (73.6)	323 (74.3)	
> 8.69	71 (4.7)	44 (4.1)	27 (6.2)	
Iron, µmol/L	12.9 ± 7.1	12.4 ± 6.6	14.1 ± 8.1	0.000
Ferritin, nmol/L	0.05 ± 0.09	0.08 ± 0.11	0.05 ± 0.05	0.053
TIBC, µmol/L	83.5 ± 18.2	82.8 ± 18.8	85.4 ± 16.5	0.012
Transferrin saturation, %	16.5 ± 9.9	16.3 ± 9.7	16.9 ± 10.3	0.301
Folate, nmol/L, *n* (%)	28.9 ± 16.6	27.6 ± 16.3	32.5 ± 17.1	0.000
< 6.8	33 (2.2)	27 (2.5)	6 (1.4)	0.000
6.8-13.4	220 (14.6)	175 (16.4)	45 (10.3)	
13.5-45.3	985 (65.6)	709 (66.5)	276 (63.5)	
> 45.3	264 (17.6)	156 (14.6)	108 (24.8)	
Vitamin B_12,_ pmol/L	232.1 ± 147.9	223.2 ± 132.6	254.1 ± 178.3	0.000

Continuous data were represented as mean ± standard deviation, while categorical data were expressed as frequencies and percentages enclosed in the parentheses. Statistical significance was assessed through the calculation of *p*-values using Student's *t* test for continuous variables and chi-square test for categorical variables. A *p*-value below 0.05 was indicated as statistical significance between insufficient and normal serum vitamin D groups. TIBC: total iron binding capacity.

**Table 2:** Associations between PCA- or RRR-derived dietary pattern and vitamin D insufficiency across the tertiles of dietary pattern scores

Dietary patterns	Tertiles (T) of dietary pattern scores (*n* = 1502)		
	T1 OR	T2 OR (95% CI)	T3 OR (95% CI)
Total population, *n*	505	486	511
Vitamin D insufficiency, *n* (%)	358 (70.9)	351 (72.2)	358 (70.1)
PCA-derived dietary pattern			
Model 1	1.00	0.91 (0.64, 1.21)	0.65 (0.49, 0.86)^**^
Model 2	1.00	0.92 (0.69, 1.23)	0.68 (0.51, 0.90)^**^
Model 3	1.00	0.92 (0.69, 1.23)	0.70 (0.52, 0.93)^*^
RRR-derived dietary pattern			
Model 1	1.00	1.52 (1.15, 2.00)^**^	1.36 (1.04, 1.78)^*^
Model 2	1.00	1.51 (1.15, 2.00)^**^	1.35 (1.03, 1.78)^*^
Model 3	1.00	1.46 (1.00, 1.95)^**^	1.17 (0.88, 1.56)

PCA-derived dietary pattern renamed as the plant and marine-based dietary pattern. RRR-derived dietary pattern renamed as the convenience food dietary pattern. Serum levels of 25(OH) vitamin D were categorized into two groups: insufficient status, defined as ≤ 75 nmol/L (≤ 30 ng/mL), and normal status, defined as > 75 nmol/L (> 30 ng/mL). Tertile 1 (T1): reference. Model 1: unadjusted. Model 2: adjusted for age and parity. Model 3: adjusted for age, parity, trimester of pregnancy, region of residence, and monthly family income. Statistical significance is denoted by * for *p* < 0.05 and ** for *p* ≤ 0.01.

**Table 3:** Association between PCA- and RRR-derived dietary patterns with serum anemia-related biomarkers

Serum variables	Dietary patterns (DP) (*n* = 1502)	
	PCA-derived DP β (95% CI)	RRR-derived DP β (95% CI)
Hemoglobin, mmol/L		
Model 1	-0.01 (-0.00, 0.00)	0.06 (0.00, 0.01)^*^
Model 2	-0.01 (-0.00, 0.00)	0.06 (0.00, 0.01)^*^
Model 3	0.00 (-0.00, 0.00)	0.04 (-0.00, 0.01)
Iron, µmol/L		
Model 1	-0.05 (-0.02, 0.00)	0.10 (0.02, 0.05)^***^
Model 2	-0.05 (-0.02, 0.00)	0.09 (0.01, 0.05)^***^
Model 3	-0.04 (-0.02, 0.00)	0.08 (0.05, 0.24)^**^
Ferritin, nmol/L		
Model 1	-0.06 (-0.28, -0.02) ^*^	0.10 (0.22, 0.72)^***^
Model 2	-0.06 (-0.28, -0.02) ^*^	0.09 (0.20, 0.70)^***^
Model 3	-0.03 (-0.20, 0.05)	0.04 (-0.05, 0.44)
TIBC, µmol/L		
Model 1	0.08 (0.01, 0.06) ^**^	-0.17 (-0.18, -0.10)^***^
Model 2	0.09 (0.01, 0.06) ^**^	-0.17 (-0.18, -0.10)^***^
Model 3	0.02 (-0.01, 0.02)	-0.05 (-0.44, -0.07)^**^
Transferrin saturation, %		
Model 1	0.00 (-0.01, 0.01)	0.00 (-0.02, 0.02)
Model 2	0.00 (-0.01, 0.01)	0.00 (-0.02, 0.02)
Model 3	0.01 (-0.01, 0.01)	-0.00 (-0.03, 0.02)
Folate, nmol/L		
Model 1	0.02 (-0.01, 0.03)	0.02 (-0.02, 0.06)
Model 2	0.00 (-0.02, 0.02)	0.02 (-0.03, 0.05)
Model 3	0.03 (-0.01, 0.03)	-0.02 (-0.05, 0.02)
Vitamin B_12,_ pmol/L		
Model 1	-0.03 (-0.26, 0.08)	0.04 (-0.11, 0.58)
Model 2	-0.04 (-0.30, 0.06)	0.03 (-0.14, 0.55)
Model 3	-0.01 (-0.21, 0.14)	-0.01 (-0.40, 0.29)

PCA derived dietary pattern renamed as plant and marine based dietary pattern. RRR derived dietary pattern renamed as convenience food dietary pattern. The values of β and the data enclosed in the parentheses represent regression coefficients and 95% confidence intervals (CIs) following the adjustment for covariates in various models. Model 1: unadjusted. Model 2: adjusted for age and parity. Model 3: adjusted for age, parity, trimester of pregnancy, region of residence, and monthly family income. Statistical significance is denoted by * for *p* < 0.05, ** for *p* ≤ 0.01, and *** for *p* ≤ 0.001. DP: dietary pattern; TIBC: total iron binding capacity.

**Table 4:** Associations of PCA-derived dietary pattern with serum anemia-related biomarkers across the tertiles of dietary pattern scores

Serum variables	Tertiles (T) of PCA-derived dietary pattern scores (*n* = 1502)		
	T1 (*n* = 505) OR	T2 (*n* = 486) OR (95% CI)	T3 (*n* = 511) OR (95% CI)
Hemoglobin, mmol/L			
Model 1	1.00	1.61 (0.52, 4.96)	1.34 (0.46, 3.89)
Model 2	1.00	1.63 (0.53, 5.02)	1.36 (0.48, 1.89)
Model 3	1.00	1.88 (0.55, 6.36)	1.25 (0.42, 3.70)
Iron, µmol/L			
Model 1	1.00	1.01 (0.78, 1.30)	1.26 (0.97, 1.62)
Model 2	1.00	1.04 (0.80, 1.35)	1.30 (1.00, 1.68)
Model 3	1.00	0.95 (0.73, 1.25)	1.18 (0.90, 1.55)
Ferritin, nmol/L			
Model 1	1.00	1.06 (0.83, 1.37)	1.41 (1.10, 1.82)^**^
Model 2	1.00	1.12 (0.87, 1.45)	1.47 (1.13, 1.91)^**^
Model 3	1.00	0.96 (0.72, 1.29)	1.16 (0.86, 1.56)
TIBC, µmol/L			
Model 1	1.00	0.93 (0.71, 1.21)	0.66 (0.50, 0.87)^**^
Model 2	1.00	0.89 (0.68, 1.16)	0.64 (0.49, 0.85)^**^
Model 3	1.00	1.07 (0.76, 1.49)	0.86 (0.61, 1.21)
Transferrin saturation, %			
Model 1	1.00	0.93 (0.72, 1.20)	0.87 (0.67, 1.12)
Model 2	1.00	0.92 (0.71, 1.18)	0.85 (0.66, 1.10)
Model 3	1.00	0.88 (0.68, 1.14)	0.84 (0.65, 1.09)
Folate, nmol/L			
Model 1	1.00	0.73 (0.53, 1.03)	0.73 (0.52, 1.03)
Model 2	1.00	0.81 (0.57, 1.16)	0.83 (0.58, 1.19)
Model 3	1.00	0.76 (0.53, 1.11)	0.70 (0.48, 1.02)
Vitamin B_12,_ pmol/L			
Model 1	1.00	0.90 (0.67, 1.21)	1.05 (0.78, 1.40)
Model 2	1.00	0.94 (0.69, 1.26)	1.08 (0.81, 1.46)
Model 3	1.00	0.86 (0.63, 1.18)	0.93 (0.69, 1.27)

PCA-derived dietary pattern renamed as plant and marine-based dietary pattern. Odds ratios (ORs) and 95% confidence intervals (CIs) are expressed for the risk of low serum biochemical variables across the tertiles of PCA-derived DP, and tertile 1 (T1) was used as the reference group. Three distinct models were executed for the binomial logistic regression analysis. Model 1: unadjusted. Model 2: adjusted for age and parity. Model 3: adjusted for age, parity, trimester of pregnancy, region of residence, and monthly family income. Variables were categorized into two tiers based on serum cutoff values: hemoglobin, 6.52 mmol/L (10.5 g/dL); iron, 10.7 µmol/L (60 µg/dL); ferritin, 0.034 nmol/L (15 ng/mL); TIBC, 43.0 µmol/L (240 µg/dL); transferrin saturation, 16%; folate, 13.6 nmol/L (6 ng/mL); and vitamin B_12_, 149.8 pmol/L (203 pg/mL). Statistical significance is denoted by ** for *p* ≤ 0.01. TIBC: total iron binding capacity.

**Table 5:** Associations of RRR-derived dietary pattern with serum anemia-related biomarkers across the tertiles of dietary pattern scores

Serum variables	Tertiles (T) of RRR-derived dietary pattern scores (*n* = 1502)		
	T1 (*n* = 505) OR	T2 (*n* = 486) OR (95% CI)	T3 (*n* = 511) OR (95% CI)
Hemoglobin, mmol/L			
Model 1	1.00	1.68 (0.40, 7.06)	1.68 (0.40, 7.06)
Model 2	1.00	1.67 (0.40, 7.06)	1.68 (0.40, 7.10)
Model 3	1.00	2.05 (0.51, 8.32)	0.70 (0.24, 2.05)
Iron, µmol/L			
Model 1	1.00	0.72 (0.54, 0.96)^*^	0.76 (0.57, 1.02)
Model 2	1.00	0.74 (0.55, 0.99)^*^	0.79 (0.59, 1.06)
Model 3	1.00	0.83 (0.64, 1.08)	0.84 (0.64, 1.09)
Ferritin, nmol/L			
Model 1	1.00	0.72 (0.54, 0.96)^*^	0.61 (0.45, 0.81)^**^
Model 2	1.00	0.74 (0.55, 1.00)^*^	0.61 (0.46, 0.83)^**^
Model 3	1.00	0.94 (0.70, 1.26)	0.91 (0.68, 1.22)
TIBC, µmol/L			
Model 1	1.00	1.51 (1.09, 2.10)^*^	1.74 (1.26, 2.41)^**^
Model 2	1.00	1.45 (1.04, 2.02)^*^	1.71 (1.23, 2.37)^**^
Model 3	1.00	1.42 (0.95, 2.14)	1.25 (0.84, 1.87)
Transferrin saturation, %			
Model 1	1.00	1.19 (0.90, 1.59)	1.10 (0.83, 1.47)
Model 2	1.00	1.19 (0.89, 1.58)	1.09 (0.81, 1.45)
Model 3	1.00	1.21 (0.90, 1.62)	1.06 (0.79, 1.42)
Folate (nmol/L)			
Model 1	1.00	0.88 (0.60, 1.29)	1.04 (0.71, 1.51)
Model 2	1.00	0.92 (0.61, 1.39)	1.13 (0.76, 1.69)
Model 3	1.00	0.98 (0.64, 1.51)	1.43 (0.93, 2.18)
Vitamin B_12_ (pmol/L)			
Model 1	1.00	0.92 (0.66, 1.30)	1.00 (0.71, 1.40)
Model 2	1.00	0.96 (0.68, 1.35)	1.04 (0.74, 1.46)
Model 3	1.00	1.01 (0.71, 1.44)	1.25 (0.87, 1.78)

RRR derived dietary pattern renamed as convenience food dietary pattern. Odds ratios (ORs) and 95% confidence intervals (CIs) are expressed for the risk of low serum biochemical variables across the tertiles of RRR-derived DP, and tertile 1 (T1) was used as the reference group. Three distinct models were executed for the binomial logistic regression analysis. Model 1: unadjusted. Model 2: adjusted for age and parity. Model 3: adjusted for age, parity, trimester of pregnancy, region of residence, and monthly family income. Variables were categorized into two tiers based on serum cutoff values: hemoglobin, 6.52 mmol/L (10.5 g/dL); iron, 10.7 µmol/L (60 µg/dL); ferritin, 0.034 nmol/L (15 ng/mL); TIBC, 43.0 µmol/L (240 µg/dL); transferrin saturation, 16%; folate, 13.6 nmol/L (6 ng/mL); and vitamin B_12_, 149.8 pmol/L (203 pg/mL). Statistical significance is denoted by * for *p* < 0.05 and ** for *p* ≤ 0.01. TIBC: total iron binding capacity.

## Abbreviations

BMI: body mass index
CFDP: convenience food dietary pattern
CI: confidence interval
DP: dietary pattern
OR: odds ratio
PCA: principal component analysis
PMBDP: plant marine-based dietary pattern
RRR: reduced rank regression
sFFQ: semi-quantitative food frequency questionnaire
TIBC: total iron-binding capacity
