# Supplementary material for: Comparative analysis of two analytical methodologies for deriving dietary patterns associated with vitamin D insufficiency and anemia among expectant mothers
Source: Int J Med Sci. 2026 Jan 1;23(1):325–33. doi: 10.7150/ijms.124006 (PMC12702124; doi:10.7150/ijms.124006)
Supplement: Supplementary file 1 — Supplementary figures and table. [file ijmsv23p0325s1.pdf]

Supplementary Table 1. General characteristics of the expectant mothers across different levels of serum vitamin D

Variables	Total population (*n* = 1502)	Insufficient vitamin D ≤ 75 nmol/L (*n* = 1067)	Normal vitamin D > 75 nmol/L (*n* = 435)	*p*
Sociodemographic and other characteristics				
Age, years, *n*	1502	1067	435	
Mean ± standard deviation	32.5 ± 4.8	32.3 ± 4.7	33.1 ± 4.8	0.500
Region of residence, *n* (%)	1499	1065	434	
Northern	501 (33.4)	408 (38.3)	93 (21.4)	0.000
Central	371 (24.8)	268 (25.2)	103 (23.7)	
Southern	291 (19.4)	141 (13.2)	150 (34.6)	
Eastern and others	336 (22.4)	248 (23.3)	88 (20.3)	
Parity, *n* (%)	1497	1064	433	
1	824 (55.0)	611 (57.4)	213 (49.2)	0.006
2	527 (35.2)	361 (33.9)	166 (38.3)	
> 3	146 (9.8)	92 (8.7)	54 (12.5)	
Trimester, *n* (%)	1502	1067	435	
First, weeks 0-12	375 (25.0)	308 (28.9)	67 (15.4)	0.000
Second, weeks 13-26	485 (32.3)	353 (33.1)	132 (30.3)	
Third, weeks 27-40	642 (42.7)	406 (38.1)	236 (54.3)	
Education, *n* (%)	1493	1061	432	
Junior high school or below	237 (15.9)	158 (14.9)	79 (18.3)	0.169

College or university	1025 (68.6)	731 (68.9)	294 (68.0)	
Graduate school	231 (15.5)	172 (16.2)	59 (13.7)	
Monthly family income, NTD, *n* (%)	1474	1049	425	
< 30,000	212 (14.4)	146 (13.9)	66 (15.5)	0.023
30,000-59,999	634 (43.0)	430 (41.0)	204 (48.0)	
60,000-99,999	443 (30.1)	331 (31.6)	112 (26.4)	
> 100,000	185 (12.5)	142 (13.5)	43 (10.1)	
Sun exposure, min/d, *n* (%)	1491	1057	434	
< 10	453 (30.4)	315 (29.8)	138 (31.8)	0.527
10 to < 20	449 (30.1)	325 (30.7)	124 (28.6)	
20 to < 60	509 (34.1)	356 (33.7)	153 (35.3)	
> 60	80 (5.4)	61 (5.8)	19 (4.4)	
Anthropometric data				
Pre-pregnancy BMI, kg/m^2, *n* (%)	1495	1061	434	
Mean ± standard deviation	22.7 ± 4.0	22.6 ± 4.0	22.9 ± 4.0	0.815
< 18.5	141 (9.4)	105 (9.9)	36 (8.3)	0.464
18.5 to < 24.0	914 (61.1)	654 (61.6)	260 (59.9)	
24.0 to < 27.0	239 (16.0)	161 (15.2)	78 (18.0)	

Continuous data were represented as mean ± standard deviation, while categorical data were expressed as frequencies and percentages enclosed in the parentheses. Statistical significance was assessed through the calculation of *p*-values using Student's *t* test for continuous variables and chi-square test for categorical variables. A *p*-value below 0.05 was indicated as statistical significance between insufficient and normal serum vitamin D groups. NTD: new Taiwan dollar; BMI: body mass index.

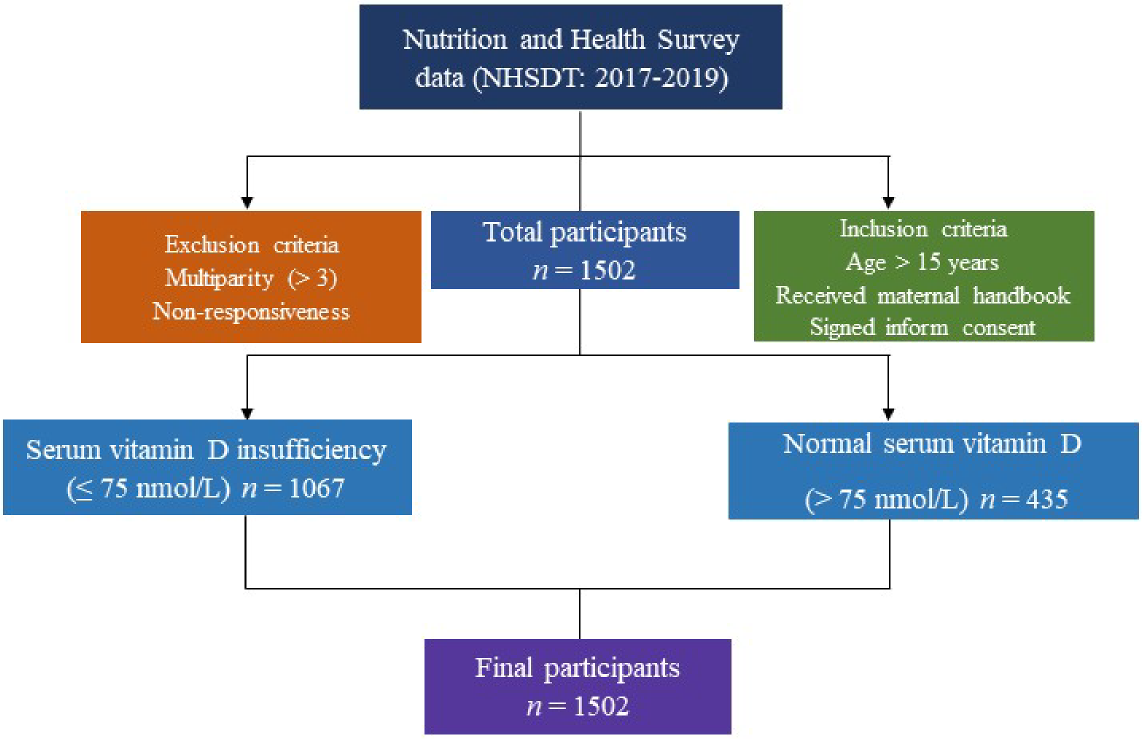

Supplementary Figure 1. Schematic diagram of the study population selection.

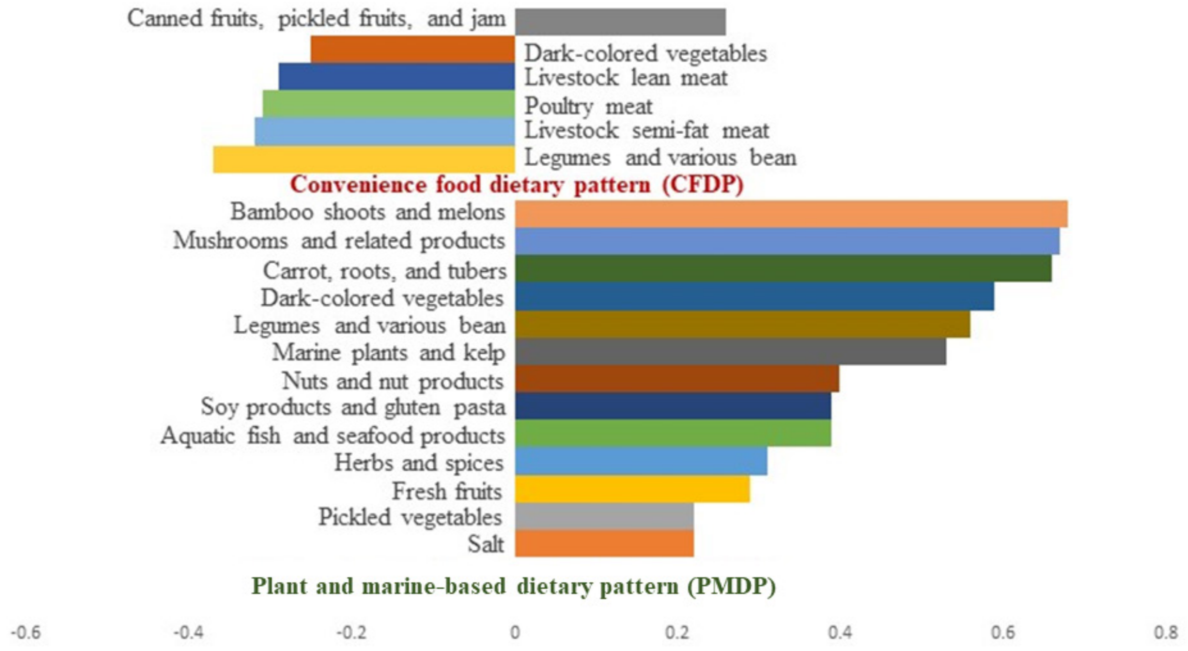

Supplementary Figure 2. Absolute factor loadings (≥ 0.20) of two dietary patterns for plant and marine-based dietary pattern (PMDP) and convenience food dietary pattern (CFDP), respectively, derived from principal component analysis and reduced rank regression analysis.
